# Supplementary material for: Coulomb spin liquid in anion-disordered pyrochlore Tb2Hf2O7
Source: Nat Commun. 2017 Oct 12;8:892. doi: 10.1038/s41467-017-00905-w (PMC5638941; doi:10.1038/s41467-017-00905-w)
Supplement: Supplementary file 1 — Supplementary Information [file 41467_2017_905_MOESM1_ESM.pdf]

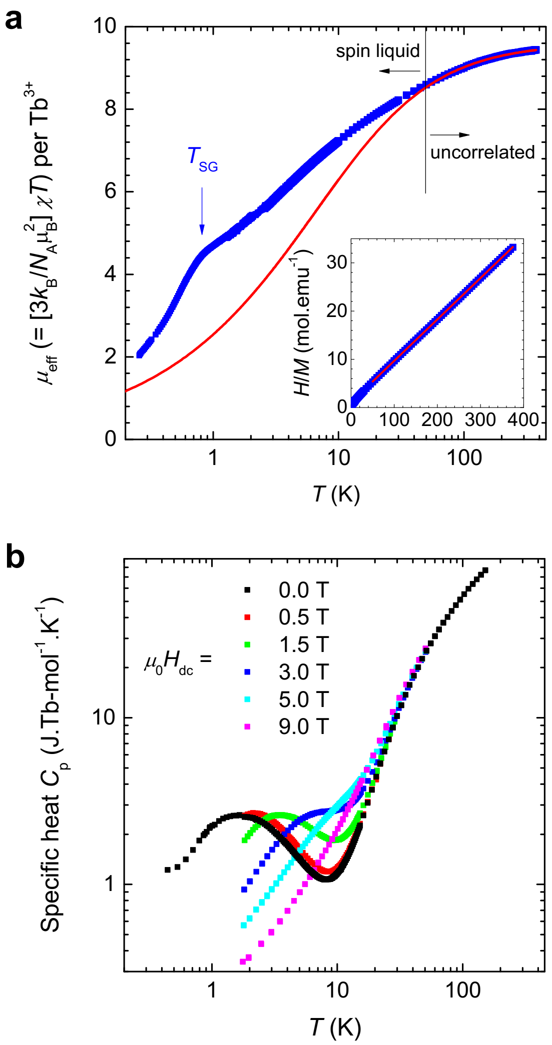

Supplementary Figure 1 | Magnetic correlations in $Tb_2Hf_2O_7$ from macroscopic measurements. a, The inset shows the applied magnetic field (*H*) divided by the magnetization (*M*), equal to the inverse susceptibility χ^{-1} in the linear field regime, plotted as a function of the temperature *T*. $\chi^{-1}(T)$ varies linearly with temperature between $T = 50$ K and $T = 375$ K. A fit to the Curie-Weiss law (red line) yields a magnetic moment $\mu = 9.60(2)$ μ_B per Tb^{3+}, in good agreement with the expected free ion value of 9.72 μ_B per Tb^{3+}. The Curie-Weiss temperature is $\theta_{CW} \approx -12$ K, suggesting antiferromagnetic interactions of the same order as for the other terbium pyrochlores. Below $T = 50$ K, $\chi^{-1}(T)$ deviates from a straight line, probably due to the onset of magnetic correlations and the depopulation of crystal-field levels. In the main panel the same data are converted to a plot of the effective magnetic moment on a logarithmic temperature scale. The red curve is the Curie-Weiss fit obtained from $\chi^{-1}(T)$ shown in the inset. The anomaly at $T_{SG} \sim 0.75$ K corresponds to the spin-glass transition. **b**, Specific heat C_p vs. *T* of a polycrystalline sample showing a hump attributed to magnetic correlations and reminiscent of $Tb_2Ti_2O_7$, which moves upwards upon increasing the applied magnetic field. The temperature dependence of the specific heat shows a broad peak centered around $T = 2$ K and no sign for a phase transition down to $T = 0.3$ K. This suggests the onset of magnetic correlations below $T = 8$ K that do not develop long-range order. Supplementary Fig. 1b also shows that magnetic fields up to $\mu_0 H = 9$ T suppress completely these magnetic fluctuations and lead to a different ground state.

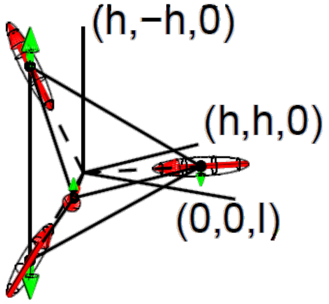

Supplementary Figure 2 | Experimental coordinates for polarization analysis on D7. We used a typical geometry for neutron polarization analysis on cubic pyrochlore magnets. The figure illustrates a tetrahedron of the pyrochlore lattice and the polarization components measured in Figs. 3**c** and 3**d**. The horizontal plane contains the wave vectors of type (h,h,l). The red and green spin components represent "2-in-2-out" and ''2-up-2-down'' ice-rule configurations, respectively. The experiment maps reciprocal space in two polarization channels, *z* (non-spin flip) and *z'* (spin flip), which allows to distinguish correlations among spin components that are perpendicular or within the (h,h,l) scattering plane, respectively.

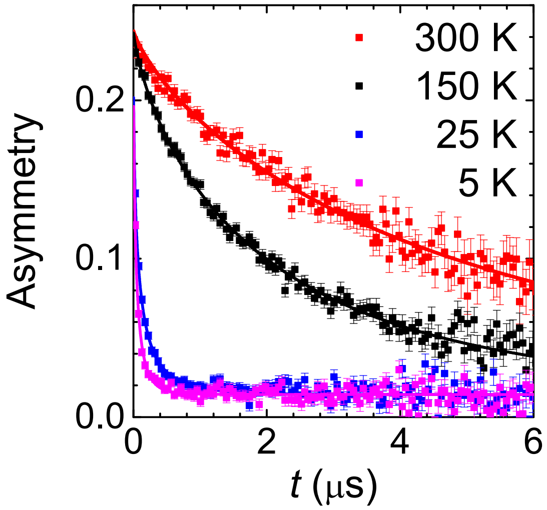

Supplementary Figure 3 | Muon spin relaxation spectra measured on a powder sample of $Tb_2Hf_2O_7$ at different temperatures. We have studied the magnetic dynamics in $Tb_2Hf_2O_7$ using muon spin relaxation in the temperature range from 0.02 K to 200 K using the instruments GPS and LTF. The experimental data used to produce the results presented on Fig. 4**c** are shown here, for a few temperatures. The time (t) histograms $A(t) = A_0 P(t)$ represent the time dependence of the polarization P of the muon spins that are stopped in the sample. The asymmetry $A(t)$ equals $[N_F(t) - \alpha N_B(t)] / [N_F(t) + \alpha N_B(t)]$, where N_F and N_B are the number of events (positrons detected) in the forward (with respect to muon spin) and backward detectors, respectively, and the parameter α is instrumental and arises from the difference of the solid angles and efficiency between the detectors. The asymmetry at time $t = 0$, A_0, is a parameter determined experimentally, which depends on instrumental factors. The data points with error bars are the experimental data while the lines are the results of fits using the stretched exponential function $A(t) = A_0 \exp[-(t/T_1)^{\beta}] + A_{bg}$, where $\lambda = 1/T_1$ is the relaxation rate. The error bars represent statistical errors determined by the number of total muon decay events observed for each data point.

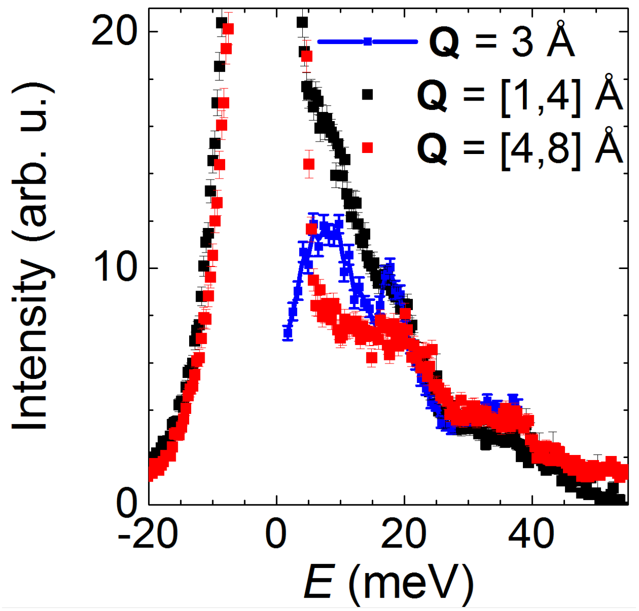

Supplementary Figure 4 | Distribution of crystal electric-field states in $Tb_2Hf_2O_7$. In order to evaluate the crystal-field spectrum of Tb^{3+} ions in $Tb_2Hf_2O_7$, we have recorded inelastic neutron scattering spectra on a powder sample. We attribute the apparent absence of discrete crystal electric-field levels to the distribution of crystal field environments around Tb^{3+}, which instead generates broad inelastic signals, mostly between 5 and 25 meV. The black and red curves show energy cuts, integrated over two different ranges of momentum transfer, through an inelastic neutron scattering spectrum measured on the time-of-flight spectrometer MERLIN at T = 7 K, using an incident energy E_i = 70 meV. The blue points show data measured at T = 1.5 K for one scattering vector, using the triple-axis spectrometer EIGER. The error bars represent statistical errors determined by the number of neutron counts for each data point.
